# Efficacy and Safety of Novel Beta-Chitin Patches as Haemostat in Rat Vascular and Neurosurgical Model

**DOI:** 10.3389/fsurg.2022.830364

**Published:** 2022-04-08

**Authors:** Ahad Sabab, Rajan Sundaresan Vediappan, John Finnie, C. John McAdam, Alistair Jukes, Sarah Vreugde, Peter-John Wormald

**Affiliations:** ^1^Department of Surgery-Otorhinolaryngology, Head and Neck Surgery, University of Adelaide, Adelaide, SA, Australia; ^2^Discipline of Anatomy and Pathology, Adelaide Medical School, University of Adelaide, Adelaide, SA, Australia; ^3^Department of Chemistry, University of Otago, Dunedin, New Zealand

**Keywords:** chitin, p-GlcNAc, haemostat, haemostasis, neurosurgery, bio-technology

## Abstract

**Background:**

Intraoperative hemorrhage is a major cause of poor post-operative outcome. Beta-chitin patch has previously been found to be an effective haemostat, but whether modifying the patch can improve its efficacy and safety, remains unknown. In this study, beta-chitin patches were modified using polyethylene oxide, Pluronic-F127 (Chi/F127), calcium (Chi/20%Ca), increased thickness (Chi/Thick) or polyphosphate (Chi/PP).

**Objective:**

Using rat (Wistar Albino; 8–10 weeks old) vascular and neurosurgical models, this project investigated and compared the efficacy and safety of beta-chitin patches with gauze, Surgicel and FloSeal.

**Methods:**

Ninety rats underwent a standardized femoral artery injury and were randomized to receive either beta-chitin patches, gauze, Surgicel or FloSeal. The bleeding time and total blood loss was measured. For the neurosurgical model, forty-four rats underwent a standardized cortical injury and randomization to a treatment group. Following a 48 h recovery period, their brains were collected for histopathological examination.

**Results:**

The mean bleeding time with Chitin (120.8 s) and Chi/PP (117.3 s) was ~60 s lower than Chi/F127, Chi/20%Ca and Chi/Thick (*p* < 0.05). Chitin and Chi/PP had a significantly lower bleeding time than FloSeal (174.2 s) (*p* < 0.05), but not Surgicel (172.7 s). Gauze (400 s) had a significantly higher bleeding time compared to all other groups (*p* < 0.05). There were no significant differences in the total blood loss between the groups. Histopathological examination of brains found no adverse inflammatory reaction to any of the haemostatic compounds.

**Conclusion:**

Chi/PP had superior haemostatic efficacy compared to Surgicel and FloSeal, but not compared to non-modified beta-chitin patch. All of the haemostats were equally safe.

## Introduction

Intraoperative hemorrhage is a major cause of poor post-operative outcome. This is especially the case in the endoscopic and neurosurgical setting, where intraoperative hemorrhage can affect the surgeon's field of vision and thereby increase the risk of surgical complications. Furthermore, intraoperative hemorrhage increases the risk of post-operative haematoma and in the fixed confines of the cranial cavity, it can have detrimental effects on the outcome of the patient.

Currently, the surgeon has a myriad of haemostatic agents and techniques in their arsenal. Techniques such as mechanical compression and electrocautery are both very effective in controlling bleeds, however, in the context of endoscopic and neurosurgical settings, these techniques are both difficult to employ, increase the risk of complete occlusion of the lumen and pose the risk of damage to the surrounding tissue (1, 2). Furthermore, while these techniques can be used to successfully control medium and large vessel bleeds, they are often inadequate in managing small vessel bleeds and diffuse tissue ooze, as is often seen in the endoscopic and neurosurgical setting (3).

Along with haemostatic techniques, there also exists a wide variety of topical haemostatic agents that have been found to be effective in controlling surgical bleeds. Examples of popular Food and Drug Administration (FDA) approved topical haemostatic agents include gelatine sponge (Gelfoam), gelatine-thrombin matrix sealant (FloSeal), microfibrillar collagen (Avitene), oxidized regenerated cellulose (Surgicel) and fibrin sealants (Tisseel) (2). However, these agents are not without side effects. For example, Gelfoam, FloSeal, Avitene and Surgicel, are all likely to swell on contact with bodily fluids, thereby increasing the risk of compression of the surrounding neural tissue, Avitene is ineffective in patients with thrombocytopenia and Tisseel requires 5–15 min to thaw and prepare, rendering them inconvenient in the case of emergency (2).

In recent years, chitin has gained popularity as a potential haemostatic agent with a good safety and efficacy profile. Chitin is a naturally occurring biopolymer that can be found in the exoskeletons of arthropods and cell walls of fungi. It is constructed from poly-N-acetyl glucosamide (p-GlcNAc) molecular chains. Deacetylation of chitin's amide functional groups generates the polyglucosamine chitosan. Both chitin and chitosan have similar properties and they are both acclaimed in literature for possessing antimicrobial (4, 5), antiadhesive (6, 7), wound healing (8, 9) and haemostatic properties (10, 11). This makes them ideal for biomedical application.

P-GlcNAc polymers have been demonstrated to promote platelet aggregation (12) and erythrocyte aggregation (13). Furthermore, the platelet-erythrocyte plug uptakes the surrounding vasodilatory products (11), such as nitric oxide, while simultaneously promoting the release of products that promote vasoconstriction (14), such as thromboxane, resulting in a net vasoconstriction, further accelerating the haemostatic process. Furthermore, the lack of dependency of the P-GlCNAc polymers on the coagulation cascade system, makes them an optimal haemostatic agent in the setting of coagulopathies and anticoagulation (11, 15, 16).

In literature, three different forms of chitin have been described, namely alpha-, beta- and gamma-chitin. The difference between the different forms of chitin depends on the orientation of their polyglucosamide molecular chains (17). Alpha-chitin has anti-parallel chain arrangement, beta-chitin has parallel polymer chains and gamma-chitin has a combination of parallel and anti-parallel chain motifs (17). There is a paucity of literature on gamma-chitin and its properties, hence it is not considered in this paper. Alpha- and beta-chitin, on the other hand, both possess excellent haemostatic properties, but research by Smith et al. has found beta-chitin to have superior haemostatic efficacy compared to chitosan and alpha-chitin (18).

The haemostatic efficacy of chitin and chitosan has been rigorously studied in literature (19–22). Studies by Brandenberg et al. and Rajiv et al. demonstrated chitosan in gel-formation to be an efficacious and safe haemostat using cat and swine models, respectively (3, 10). However, little is known about the safety and efficacy of chitin in neurosurgical setting.

Recently, through collaboration with the University of Otago, Department of Chemistry, our group has developed a novel, solid, beta-chitin patch. While *in vitro* assessment of efficacy and safety has yield promising data, the efficacy and safety of the beta-chitin patch in animal models, is unknown. Similarly, little is known about the effect of beta-chitin patch modification on its efficacy and safety in animal models.

The aim of this paper is to evaluate the efficacy and safety of modified and non-modified beta-chitin patches and compare them to the current standards of care. The beta-chitin patch modifications that was utilized for this study included incorporation of polyethylene oxide (Chi/PEO), Pluronic-F127 (Chi/F127), calcium (Chi/20%Ca), increased thickness (Chi/Thick) or polyphosphate (Chi/PP). Addition of PEO was expected to improve the flexibility and sealant ability of the patch, allowing better adhesion to the site of injury and thereby improving the haemostatic capacity (23). Pluronic-F127, a surfactant, is known to allow better dispersion of nanofibers, hence modifications with Pluronic-F127 will allow even dispersion of chitin fiber, preventing their aggregation to a singular site on the patch (24). Calcium and polyphosphate are key components in the coagulation cascade, hence patches with calcium or polyphosphate should show improved the haemostatic capacity (25, 26). Finally, increasing the thickness should allow better trapping of erythrocytes, platelet, coagulation factors, and other blood products, whilst simultaneously providing better tamponade of the bleeding vessel, minimizing blood loss and accelerating the haemostatic process.

## Methods

The University of Adelaide Animal Ethics Committees (AEC) approved the study to be conducted at The Queen Elizabeth Hospital Experimental Surgical Suite (Ethics Approval #: M-2018-124). All experiments in this study were undertaken in accordance with the ethics approval and organizational policies and guidelines.

### Novel Beta-Chitin Patch Preparation

#### Chitin Digestion and Dispersion

Dry squid pens were ground and the fraction that passed through a 250 μm sieve (Endecotts) collected. Protein was digested using 1 M NaOH and the resulting solid collected by filtration, rinsed with H_2_O till pH neutral, then ethanol and air-dried. Typical weight loss for the digestion process was 53%.

The digested chitin was suspended in acetic acid (1% v/v) and dispersed by a mechanical blender to give “Chitin”, an opaque thick suspension with no separate liquid.

#### Bilayer Patch Fabrication

All backings were prepared by filtering with suction a portion of dispersed Chitin/PEO 1000 (20 wt% of chitin) on Whatman paper to dryness. Typical backing layer dimensions: ø 42 mm × 0.01 mm, weight 26 mg (~5% PEO by wt). For the foam topping, 5.0 g of the selected chitin dispersion [one of Chitin, Chitin/PEO 1000 (20 wt% of chitin), Chitin/Pluronic F127 (20 wt% of chitin), Chitin/calcium acetate monohydrate [5743-26-0] (20 wt% of chitin), or Chitin/sodium phosphate glass (Aldrich S4379, 20 wt% of chitin)] was added to a backing layer disk in a petri dish, the combination frozen then lyophilised for >24 h. Typical bilayer patch dimensions: ø 39 mm × 2 mm, 50–55 mg. The nominal calcium loadings were 1.1 mg Ca^2+^ per patch for 20 wt% preparations.

#### Thick Bilayer Patch Fabrication

**Figure 1:**
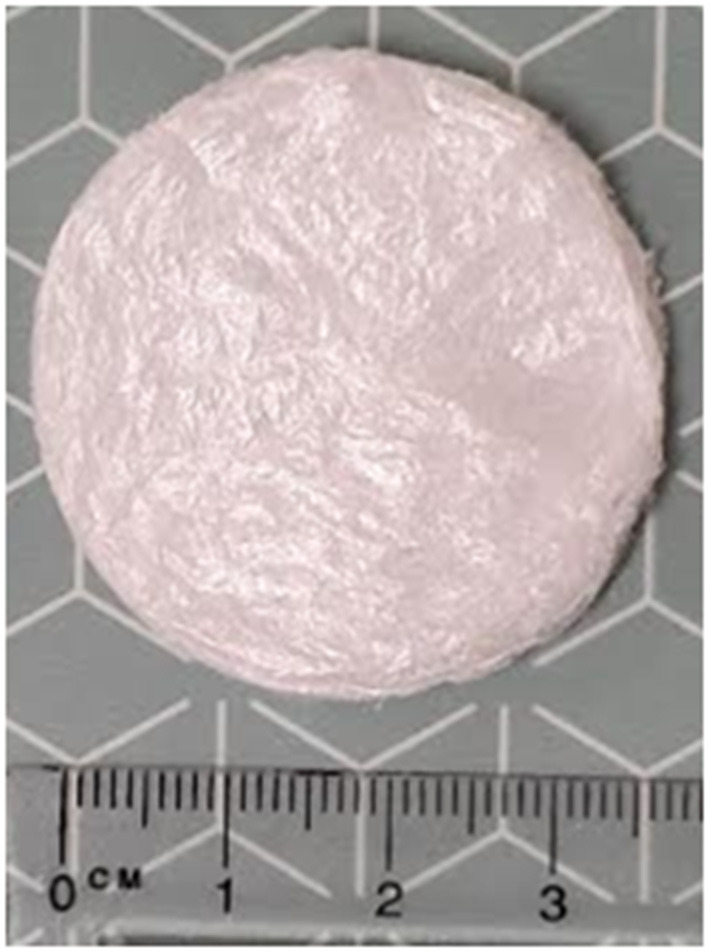
Beta-chitin patch following preparation.

Thick bilayer patches were prepared similarly from 10.0 g of the Chitin dispersion (*foam topping*). Typical thick patch dimensions: ø 39 mm × 4–5 mm, 100 mg (Figure 1).

### Rat Femoral Arterial Bleed

Ninety male Wistar Albino rats (390–440 g), aged 8–10 weeks were purchased from the laboratory animal services. After 1 week of acclimatization, the animals were randomly allocated to one of the following treatment groups: Chitin, Chi/F127, Chi/PEO, Chi/20%Ca, Chi/Thick, Chi/PP, Surgicel, FloSeal and Gauze; with ten rats per group. Following allocation, the rats were weighed and anesthetized with 3% isoflurane. After induction, the animal's paw reflexes were checked to ensure complete induction. The right inguinal region was shaved and cleaned with ethanol prior to surgery.

#### Surgical Technique

**Figure 2:**
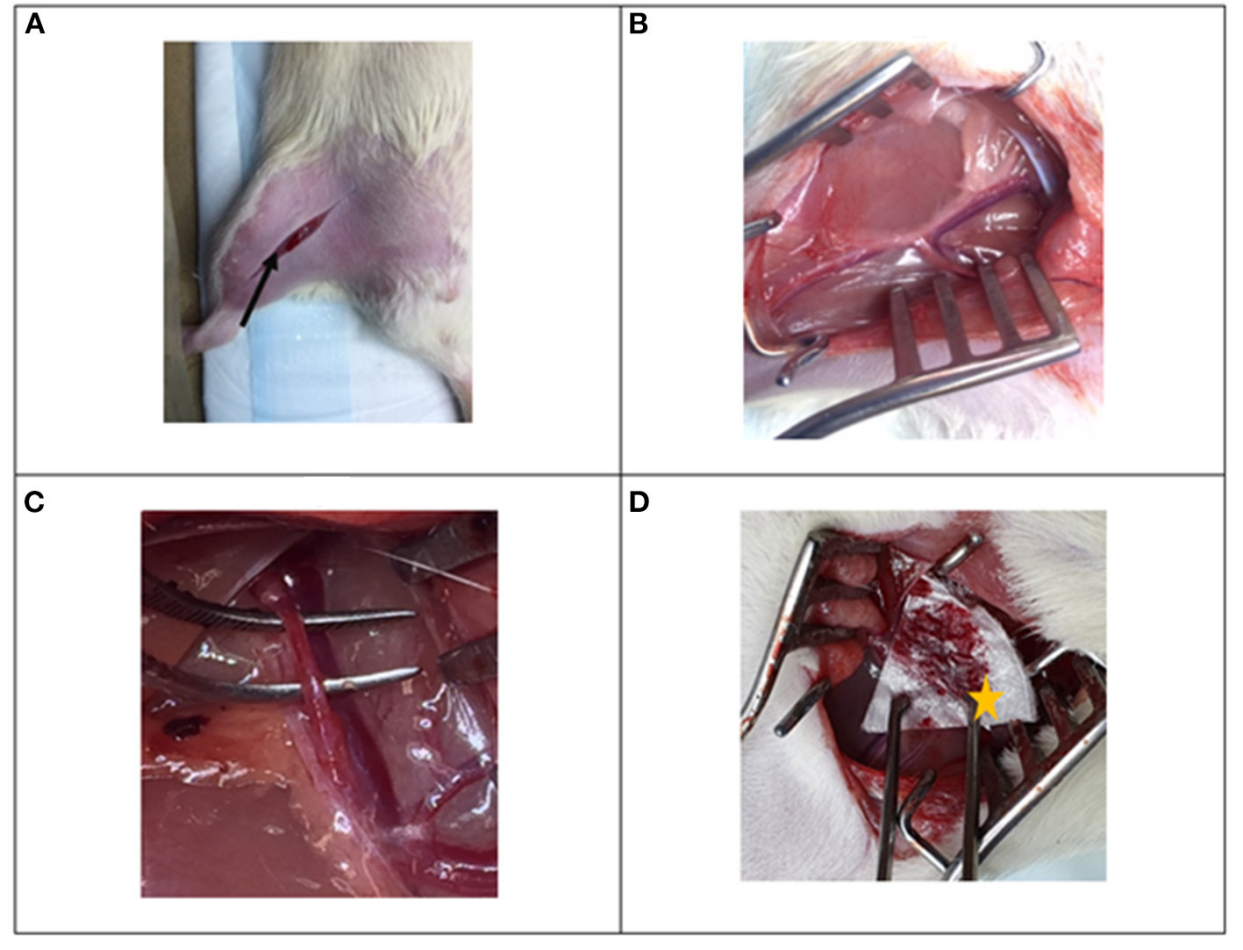
Photographs of surgical procedure for the rat femoral arterial injury experiment. **(A)** Inguinal incision is made perpendicular to the inguinal canal (black arrow), **(B)** the femoral bundle is identified and exposed and **(C)** the proximal femoral artery is dissected away from the femoral vein and femoral nerve. **(D)** An injury is made to the vessel and the beta-chitin patch (star) is applied to the injury.

An inguinal incision was made on the right leg, perpendicular to the inguinal canal (Figure 2A). The proximal femoral artery was identified (Figure 2B) and was carefully dissected away from the surrounding femoral nerve, femoral vein and fascia (Figure 2C). All instruments were moved away from the artery and a period of 1 min was allowed to relieve any vasospasm. Following this a standardized puncture was made on the anterior wall of the femoral artery using a 23-gauge needle. The artery was allowed to bleed freely for 5 s after which the test agent was applied to the site and was compressed with the weight of a large, 250 g forceps (Figure 2D). Pre-weighed gauze (*G*_*i*_) was placed around the surgical site, to absorb the blood lost. An investigator blinded to the treatment decided when the bleeding had stopped and carefully recorded the bleeding time. The used gauze was re-weighed (W_f_) and the total blood loss was calculated as, *W*__*f*_−_*W*_*i*_, and it was assumed that 1 g of blood is equivalent to 1 mL of blood. Upon completion of the experiment, the animals were humanely euthanized by pentobarbital (120 mg/kg) injection to the heart.

### Rat Surgical Brain Injury

Forty-four male Wistar Albino rats (390–440 g), aged 8–10 weeks were allocated to this study. After 1 week of acclimatization, the animals were randomly allocated either one of the 10 treatment groups or positive control. A total of four rats were allocated to each individual treatment group—Chitin, Chi/F127, Chi/PEO, Chi/20%Ca, Chi/Thick, Chi/PP, Saline, Surgicel, FloSeal and gauze, with the positive control being kaolin. Following allocation, the rats were weighed and anesthetized as previously described.

#### Surgical Technique

**Figure 3:**
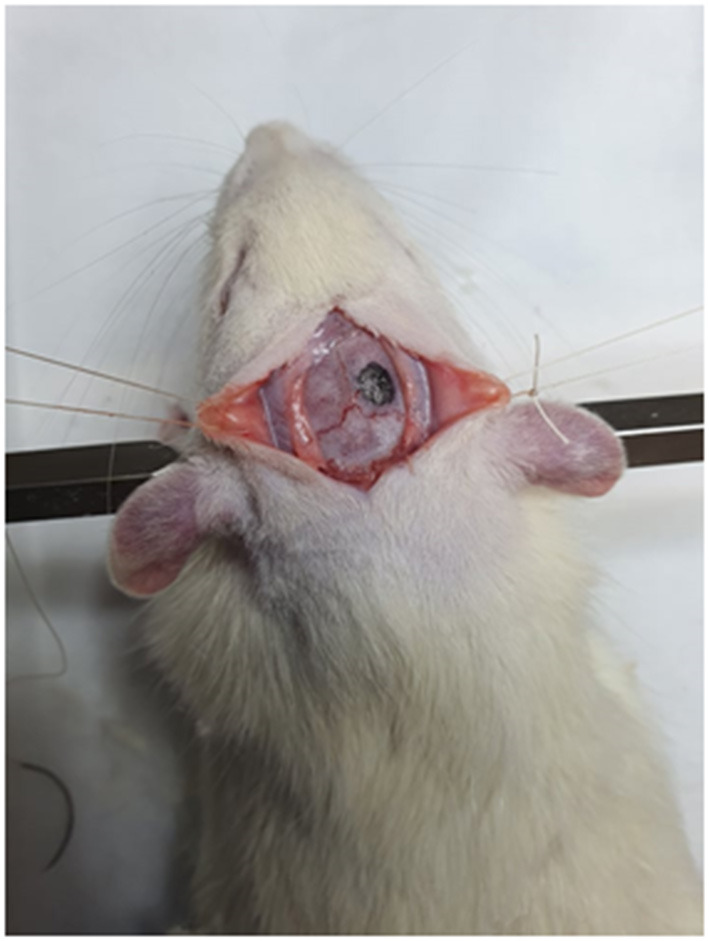
Photograph of drill site for rat neurosurgical model.

Following induction, the animals were placed prone in a stereotaxic frame, under surgical microscope. The animals were then injected with buprenorphine (0.05 mg/kg), 5 ml saline and antibiotic (amoxicillin). The head was shaved and cleaned with a combination of alcohol and iodine. A midline scalp incision was made, and the underlying periosteum bordered by the sagittal suture, bregma and lamboid suture was carefully dissected away from the bone, exposing the skull. Using a micro-drill, a 5 mm burr hole was created such that the hole was located 1 mm anterior and lateral to lamboid and sagittal suture, respectively (Figure 3). The drilling was discontinued when a fine layer of bone remained on top of the dura and it was removed using forceps to expose the dura without any injury. The dura was then carefully incised in a cruciate fashion using a 23-gauge needle and reflected. A size 11 scalpel blade was then inserted 2 mm into the brain and it was rotated to create a circular injury of 2 mm diameter. The sectioned brain tissue was removed, and, according to randomization, different treatment was applied to ensure complete coverage of the injury site. Upon cessation of cerebral bleeding, bone wax was applied over the bone window and the periosteum and skin were sutured.

The anesthetics was ceased, and the animals were removed from the stereotaxic frame, and allowed to recover under 2L oxygen. Upon recovery the animals were returned to their cages. They were monitored eight-hourly over the next 2 days, analgesics and antibiotics were provided during each monitoring. The animals were euthanized 48 h post-surgery, brain was removed and immediately immersion-fixed in 10% neutral buffered formalin. Coronal slices were then cut, paraffin-embedded, and 6 μm sections cut and stained with haematoxylin and eosin. Qualitative and quantitative histopathological examination was undertaken by two pathologists, who were blinded treatment groups.

### Statistical Analysis

All data is expressed as mean ± standard deviation. Kruskal-Wallis one-way ANOVA was utilized for statistical analysis; using Dunn's test with Benjamini-Hochberg correction to perform pair-wise comparisons. *P*-values <0.05 were considered statistically significant.

## Results

### Rat Femoral Arterial Bleed

**Figure 4:**
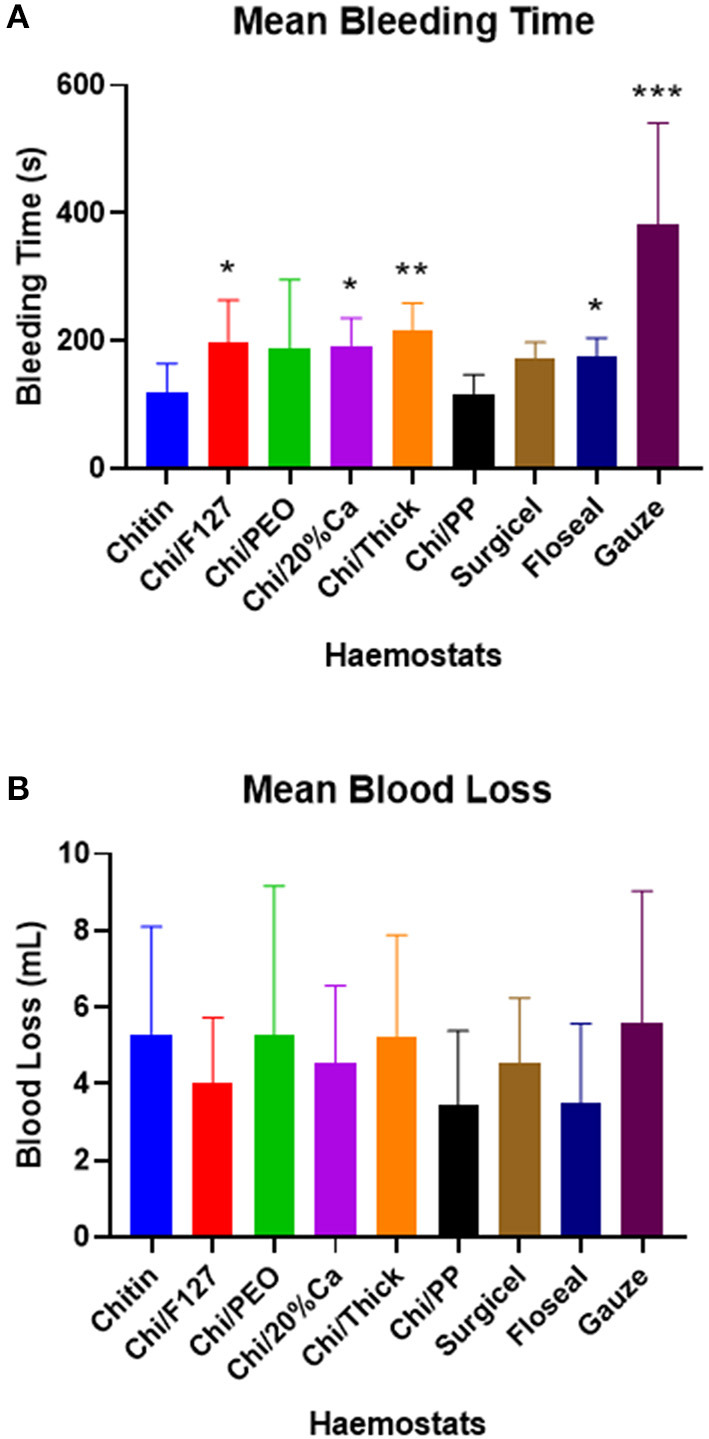
**(A)** The mean bleeding time and **(B)** the mean blood loss, with rat femoral arterial injury experiment. * *p* < 0.05, ** *p* < 0.001, compared to chitin and *** *p* < 0.05 compared to chitin (modified and non-modified), Surgicel and FloSeal, analysed by Kruskal-Wallis one-way ANOVA with Dunn's test.

Chitin and Chi/PP had a mean bleeding time of 120.8 and 117.3 s, respectively, and this was ~60 s lower than most haemostats (Figure 4A). Both Chi/F127, Chi/20%Ca and Chi/Thick took ~180 seconds to achieve haemostasis, which was significantly longer than that required by Chitin and Chi/PP (*p* < 0.05) (Figure 4A). Surgicel and FloSeal took 172.7 and 174.2 s on average to stop the bleeding, respectively. Both Chitin and Chi/PP had a significantly lower bleeding time compared to FloSeal (*p* < 0.05) (Figure 4A). Gauze was a poor haemostat, requiring almost 400 s to stop the bleeding (Figure 4A).

There were no significant differences in the total blood loss between the groups (Figure 4B).

### Rat Surgical Brain Injury—Qualitative Analysis

A full-thickness traumatic cerebral cortical wound was produced and the resulting parenchymal defect plugged with various haemostatic compounds. There was a necrotic penumbra of cortical tissue, which was characterized by homogenisation of the neuropil, pyknotic glial nuclei, and marked neuronal degeneration and necrosis, many neurons manifest as “ghost” remnants.

**Figure 5:**
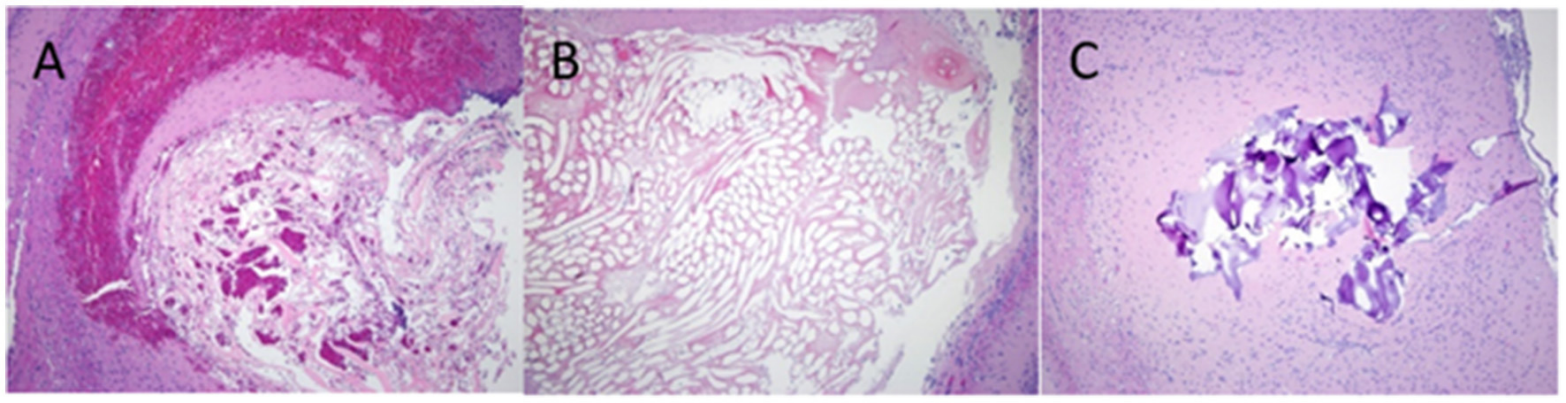
H&E staining of rat brain following cortical injury treated with, **(A)** Chitin, **(B)** Surgicel and **(C)** FloSeal.

There was no significant adverse inflammatory reaction to any of the test haemostatic compounds. The histology of chitin, Surgicel and FloSeal is shown in Figure 5, the wound hemorrhage being confined beneath the haemostatic plug, and within the interstices of the chitosan material.

### Rat Surgical Brain Injury—Quantitative Analysis

**Figure 6:**
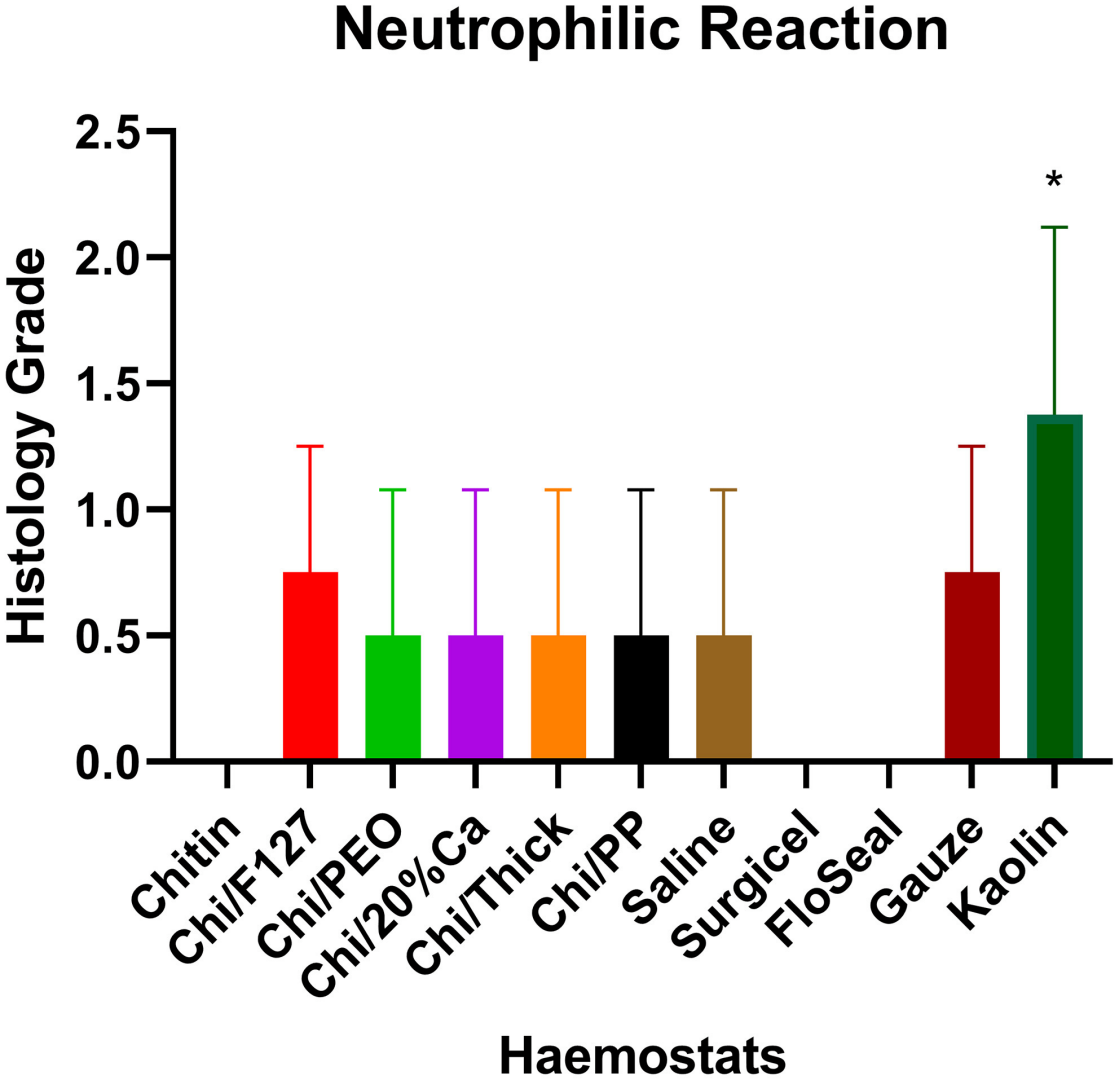
Neutrophilic reaction seen with the application of modified and non-modified beta-chitin patches, Surgicel, FloSeal, Gauze and Kaolin (positive control) to rat brain. * *p* < 0.05 and compared to non-modified beta-chitin patch, analyzed by Kruskal-Wallis one-way ANOVA with Dunn's test, *n* = 4.

**Figure 7:**
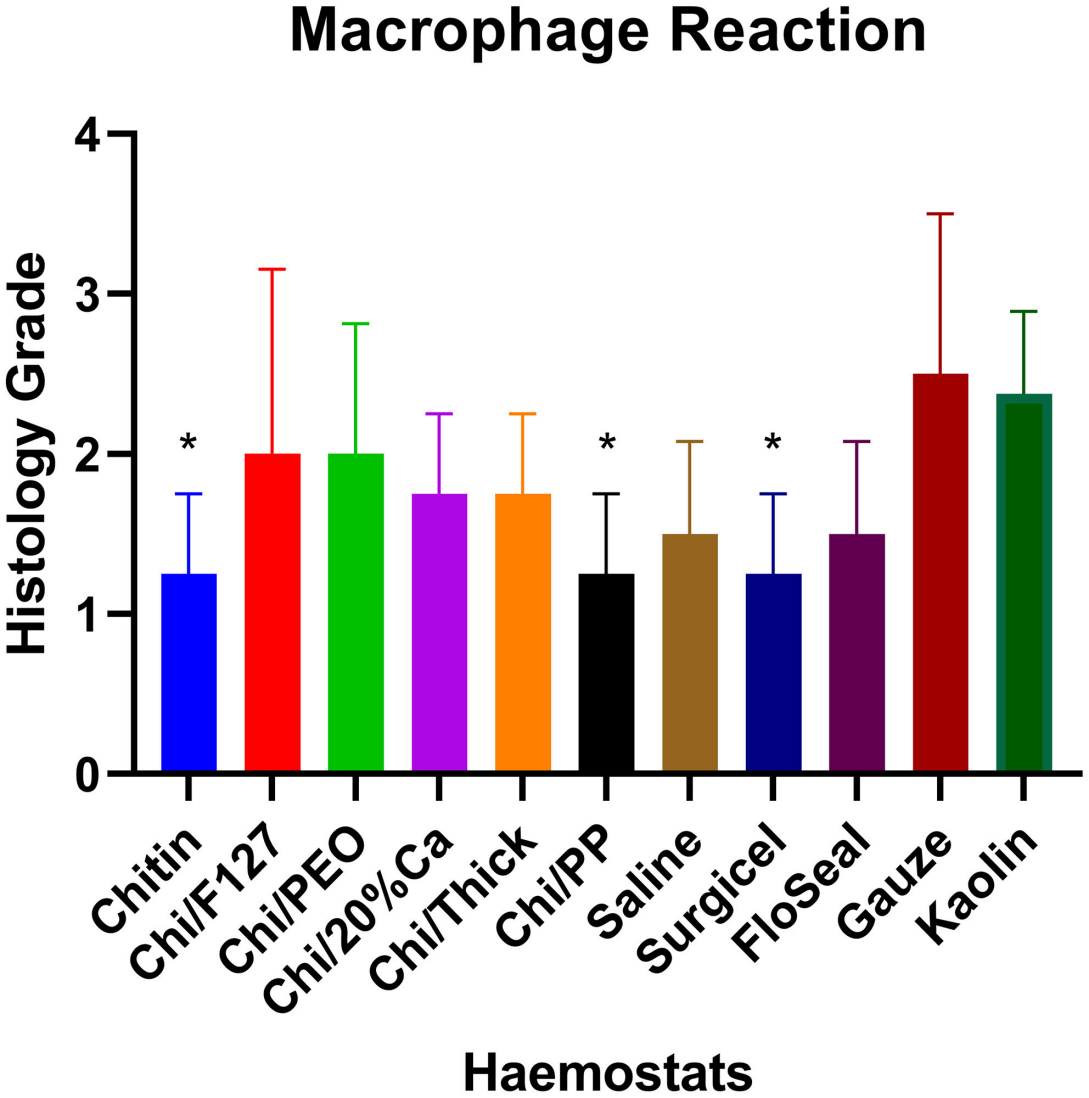
Macrophage reaction seen with the application of modified and non-modified beta-chitin patches, Surgicel, FloSeal, Gauze and Kaolin (positive control) to rat brain. * *p* < 0.05 and compared to Gauze and Kaolin, analyzed by Kruskal-Wallis one-way ANOVA with Dunn's test, *n* = 4.

**Figure 8:**
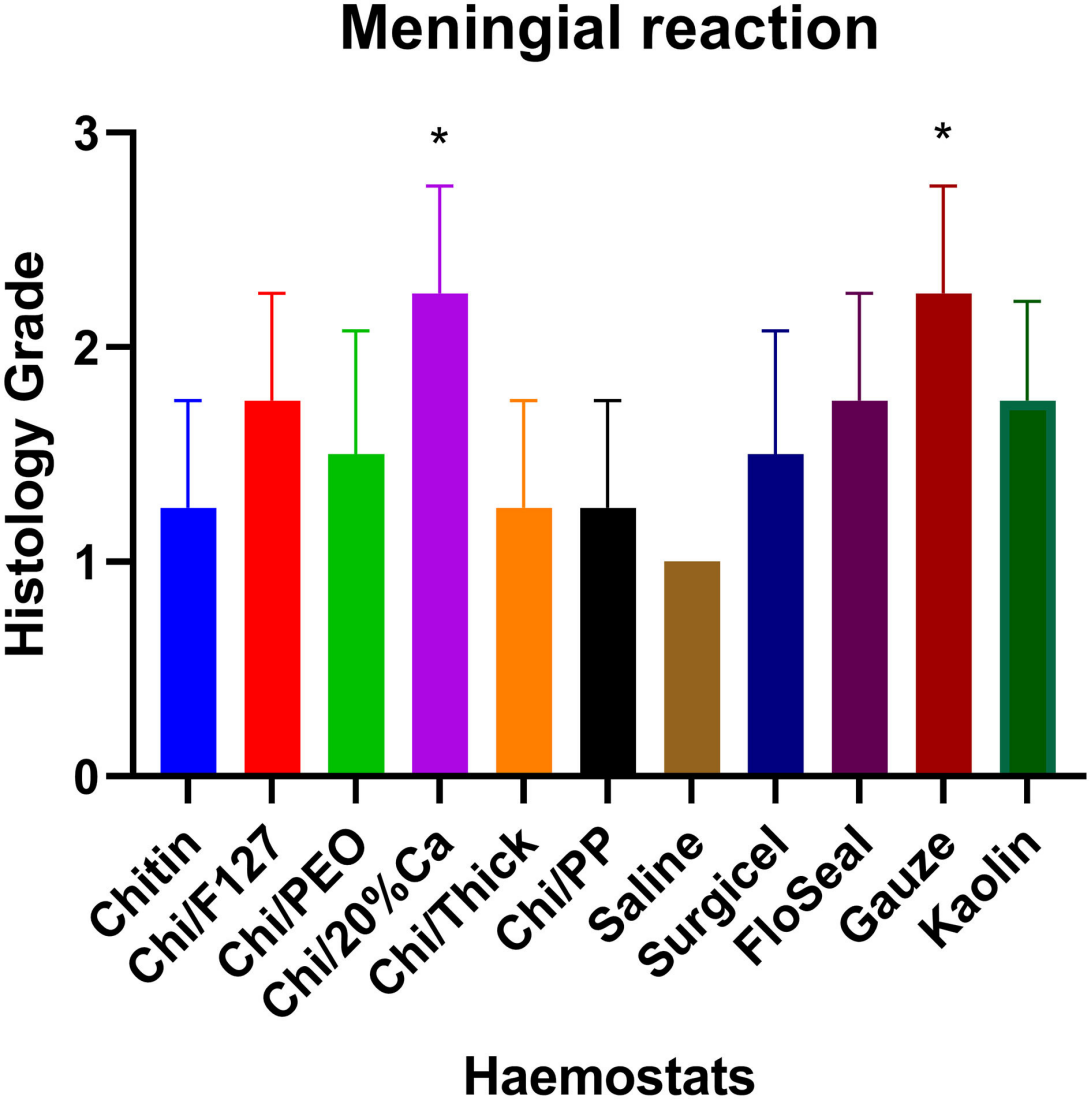
Meningeal mononuclear cell reaction with the application of modified and non-modified beta-chitin patches, Surgicel, FloSeal, Gauze and Kaolin (positive control) to rat brain. * *p* < 0.05 and compared to non-modified beta-chitin patch, analyzed by Kruskal-Wallis one-way ANOVA with Dunn's test, *n* = 4.

Quantitative histopathological analysis of the brain usually revealed a generally mild neutrophilic infiltrate, this being a probable response to necrotic brain tissue resulting from the induced wound. However, in kaolin-treated rats, the neutrophilic infiltrate was significantly greater than that for chitin, Surgicel and FloSeal (Figure 6). There was also mild to moderate macrophage infiltrate, probably elicited to both phagocytose necrotic neural debris and as a response to the foreign test material. This macrophage reaction was significantly greater with gauze and kaolin (Figure 7). In the meninges adjacent to the wound cavity in rats with implanted material, there was invariably an inflammatory infiltrate comprised of mononuclear cells (lymphocytes and macrophages) and, sometimes, a few eosinophils. Chi/20%Ca and Gauze had significantly greater meningeal reaction compared to Chitin (Figure 8).

## Discussion

This study is the first to investigate the haemostatic efficacy and safety of a beta-chitin patch and modifications thereof and compare them to the current standards of care. The results demonstrate that, overall, Chi/PP has a similar, or even improved, haemostatic property with less acute inflammatory cell profile compared to non-modified beta-chitin and standards of care, Surgicel and FloSeal in a rat femoral artery and neurosurgical model.

The rat femoral arterial experiment demonstrated that the average bleeding time for the beta-chitin patches ranged from 120 to 200 s. Similar results have also been reported by Seon et al. (27) and Crofton et al. (28), who utilized the rat femoral arterial injury experiment to assess the haemostatic efficacy of chitosan dressings. Crofton et al. and Seon et al. found the bleeding time with chitosan dressings ranged between 60–100 s and 240–300 s, respectively (27, 28). This difference in bleeding time quoted in literature and that found in the present study, may be due to a combination of variations in the haemostats and their physical/chemical modifiers, surgical technique, size of vascular injury and the strain, gender, age and weight of the rats used.

In this study, use of Chi/PP resulted in a lower bleeding time compared to non-modified chitin patches and standards of care. In particular, the bleeding time was significantly lower than that attained with the use of FloSeal (117.3 s vs. 174.2s, *p* < 0.05). This difference may be attributed to the polyphosphate within Chi/PP and the solid patch conformation of Chi/PP compared to the gel-like conformation of FloSeal. Under physiological states, platelets have been shown to secrete polyphosphate upon activation, which in turn acts on the coagulation cascade by accelerating factor V and XI activation, inhibiting the ability of tissue factor pathway inhibitor which is an inhibitor of factor Xa and promotes fibrin polymerization, thereby promoting haemostasis (26). Similarly, in the setting of spurting rat femoral arterial bleed, the gel-like nature of FloSeal made it more prone to being washed away, whilst the solid patch formation of Chi/PP allows for greater absorption of blood, which in turn increased its weight and thereby provides a better tamponade. Whilst FloSeal is an excellent haemostat, its gel-like nature makes it more suitable for diffuse ooze bleeding. Overall the formulation of the beta-chitin patch and its solids nature may potentially explain the reason for accelerated haemostasis with the use of Chi/PP.

The results of this study demonstrated that, with the exception of Chi/PP, all modified beta-chitin patches were found to have a bleeding time similar to Surgicel and FloSeal, that was ~60–90 s longer than non-modified beta-chitin patch and Chi/PP. To put this into clinical context, a small artery such as the superficial temporal artery in humans (mean diameter: 1.45 mm) has a mean blood flow volume of 0.71 mL/s (29). Hence, over the 90 s interval difference between beta-chitin patches (non-modified beta-chitin patch and Chi/PP) and standards of care (Surgicel and FloSeal), ~65mL of blood would be lost. While this level of blood loss will be insignificant in an open surgical field, in the narrow confines of endoscopic skull base surgery, this volume of blood can circulate in CSF spaces and accumulate in spaces that can not be reached with instrumentation, leading to post-operative complications such as obstructive hydrocephalus.

The results on blood loss acquired from the *in vivo* rat arterial injury model had some limitations, as evident by the large standard deviations. Firstly, the rat femoral artery has an average diameter of 0.64 ± 0.16 mm (30), hence, even minor variations in arterial injury may have a major impact on the quantity of the blood lost. Furthermore, the total blood loss was very small in each rat, ranging between 3.5 and 5.6 mL. Being such a small quantity, even small inaccuracies in the collection of blood can produce major variations within the data. Whilst the results presented in this paper are acquired under controlled laboratory settings, it is imperative to understand that in a clinical setting a variety of injuries can inadvertently be made to the artery including complete laceration. This in turn will influence the bleeding time and blood loss. However, as explained previously, in context of small vessel injury, these differences will not affect clinical outcomes in the patient.

The rat surgical brain injury results, in this study, demonstrated minimal acute and chronic inflammatory reaction with the use of modified and non-modified beta-chitin patches when applied to the brain parenchyma. These findings are consistent with those of Brandenburg et al. (10) and Rajiv et al. (3), who demonstrated that chitosan-based haemostats are safe for application to animal central nervous tissue. In their study, Brandenburg et al. and Rajiv et al. utilized a cat surgical brain injury model with 6–8-week recovery and sheep surgical brain injury model with 3 months recovery, respectively (3, 10). Following recovery, the authors assessed the extent of macrophage reaction within the brain tissue (3, 10). In both studies, the authors compared a chitosan-based haemostat with Gelfoam, with Brandenburg et al. testing further haemostats including collagen, Surgicel and Thrombin (3, 10). The authors found no significant difference in the macrophage reaction between haemostats (3, 10). Similar findings are also recorded in this study, where there was no significant difference between the beta-chitin patches and the standards of care, Surgicel and FloSeal.

The histopathological results with Surgicel and FloSeal in this study were consistent with those reported by Ereth et al. (31), who studied the inflammatory effect of Surgical and FloSeal using a rat neurosurgical model similar to that used herein (31). The authors demonstrated that, in the acute stage (6 h, 12 h, and 3 days), there was negligible difference in the inflammatory response between the two haemostatic agents (31); similar to the present study.

In the present study, all treatment arms produced a mild to moderate, mononuclear cell meningeal inflammatory infiltrate, which was not reported in any previous studies with the use of beta-chitin and standards of care. This meningeal inflammatory reaction may have been a response to the haemostats directly applied to the brain parenchyma, and to the bone wax that overlay the dura and covered the bony defect in the skull, and likely accounted for the similarity in the meningeal reaction between the groups.

This study demonstrated that beta-chitin patch, especially when modified with polyphosphate (Chi/PP) is an efficacious and safe haemostat. However, the small animal (rat) model used was a limitation of this study as there are major differences in the parameters regarding the vascular size, flow and pressure, compared to humans. Similarly, during a neurosurgical procedure, the extent of brain tissue dissection will often be larger. For this reason, further research using large animal models is required to confirm these findings.

Another limitation of this study was that the study did not investigate the efficacy of the patches in the setting of coagulopathy or anticoagulation. Whilst a myriad of studies have previously found chitin and chitosan to be an efficacious haemostat in the context of altered coagulation, as Chi/PP was the most efficacious haemostat and the haemostatic action of polyphosphate is dependent on intact coagulation pathway, further research is required to investigate the efficacy of Chi/PP in the setting of altered coagulation.

## Conclusion

In summary, this is one the first studies to investigate the efficacy and safety of novel beta-chitin patch using animal model. The results of this study indicate that beta-chitin patch modified with polyphosphate (Chi/PP) promotes haemostasis significantly more rapidly than FloSeal. Furthermore, none of the beta-chitin patches induced any significant acute or chronic inflammatory reaction when applied to the brain. While these results highlight the potential of Chi/PP as an effective haemostat, further research using large animal models is required to confirm these findings, and to obtain data that can be translated into clinical practice.

## Data Availability Statement

The raw data supporting the conclusions of this article will be made available by the authors, without undue reservation.

## Ethics Statement

The animal study was reviewed and approved by University of Adelaide Animal Ethics Committees (AEC).

## Author Contributions

AS designed and undertook the experiments and wrote the manuscript. RV and JF designed and undertook the experiments and drafted manuscript. CM undertook the formulation of the patches used for experiments and drafted manuscript. AJ, SV, and P-JW supervised the study design and drafted manuscript. All authors contributed to the article and approved the submitted version.

## Conflict of Interest

AJ and P-JW are inventors on a patent of this patch. The remaining authors declare that the research was conducted in the absence of any commercial or financial relationships that could be construed as a potential conflict of interest.

## Publisher's Note

All claims expressed in this article are solely those of the authors and do not necessarily represent those of their affiliated organizations, or those of the publisher, the editors and the reviewers. Any product that may be evaluated in this article, or claim that may be made by its manufacturer, is not guaranteed or endorsed by the publisher.
